# The Relationship between Surrogate Markers of Insulin Resistance and Occurrence of Colorectal Adenoma in Individuals under 50 Years Old: A Single-Center Retrospective Cross-Sectional Study

**DOI:** 10.3390/jpm14090971

**Published:** 2024-09-13

**Authors:** Chi Hyeon Choi, Sang Yi Moon, Jong Yoon Lee

**Affiliations:** Division of Gastroenterology, Department of Internal Medicine, Dong-A University College of Medicine, Busan 49201, Republic of Korea; hyunt23@gmail.com (C.H.C.); sang4401@dau.ac.kr (S.Y.M.)

**Keywords:** insulin resistance, colorectal neoplasms, colorectal cancer

## Abstract

(1) Background: Young-onset colorectal adenomas (YOAs) are precursors to early-onset colorectal cancer, a growing concern among individuals under 50 years old. This study investigated the association between surrogate markers of insulin resistance (IR) and YOAs occurrence. (2) Methods: A retrospective cross-sectional analysis was conducted on 4467 individuals aged 20 to 49 years who underwent their first screening colonoscopy at Dong-A University Hospital from 2018 to 2022. IR was assessed using the triglyceride–glucose (TyG) index, triglyceride-to-high-density lipoprotein cholesterol ratio (TG/HDL-C), and metabolic score for insulin resistance (METS-IR). (3) Results: Individuals with YOAs exhibited significantly higher median TyG index (8.51 ± 0.71 vs. 8.32 ± 0.61, *p* < 0.001), TG/HDL-C ratio (2.78 ± 3.05 vs. 2.12 ± 1.85, *p* < 0.001), and METS-IR (35.72 ± 8.37 vs. 33.44 ± 9.11, *p* < 0.001) values than controls. The adjusted odds ratios for YOAs were 1.064 (95% CI: 1.22–2.23, *p* = 0.021) for the TyG index, 1.067 (95% CI: 1.031–1.105, *p* < 0.001) for the TG/HDL-C ratio, and 1.011 (95% CI: 1.002–1.021, *p* = 0.023) for METS-IR values, indicating a strong association between higher IR marker values and the presence of YOAs. (4) Conclusions: Elevated IR marker values are strongly associated with the occurrence of YOAs in individuals under 50 years old.

## 1. Introduction

Colorectal cancer (CRC) is one of the most common cancers worldwide and is a leading cause of cancer-related deaths [1]. Approximately two-thirds of those diagnosed with CRC do not have a family history or inherited genetic mutations that increase their risk [2]. Factors associated with sporadic CRC include modifiable risk factors, such as obesity, a Western diet, insufficient physical activity, alcohol consumption, and smoking. Additionally, metabolic syndrome (MetS) is identified as a group of metabolic disorders that significantly increases the risk of CRC [3,4]. Insulin resistance (IR), a central feature of metabolic disorder, is a condition in which cells in the muscles, fat, and liver start responding poorly to insulin and cannot easily take up glucose from the blood. The hyperinsulinemic–euglycemic glucose clamp technique is acknowledged as the gold standard for quantifying IR [5]. However, surrogate IR markers have been developed to simplify the labor-intensive and challenging measurements of these metrics. The triglyceride–glucose index (TyG index) is an indicator used to identify IR, which can be measured based on fasting blood glucose (FBG) and triglyceride (TG) levels. The TyG index has also been reported to be an indicator that can predict the occurrence of type 2 diabetes and cardiovascular diseases [6,7]. Accordingly, some studies have reported the association between this index and CRC [8,9]. Additionally, similar indicators, such as the TG-to-high-density lipoprotein cholesterol (TG/HDL-C) ratio and the metabolic score for IR (METS-IR) have also been used as similar markers and have been reported to be associated with CRC [10,11].

Despite significant advancements in screening and treatment, the incidence of early-onset colorectal cancer (EOCRC), defined as CRC diagnosed in individuals younger than 50 years, has been increasing alarmingly [12]. Studies indicate that EOCRC has distinct genetic features compared to late-onset CRC [13]. For example, hereditary syndromes, such as Lynch syndrome, are prevalent among EOCRC cases, but environmental and lifestyle factors, such as diet, obesity, and sedentary behavior, also play critical roles in EOCRC development [14,15]. The aim of this study was to investigate the association between these surrogate markers of IR and the occurrence of young-onset adenomas (YOAs), which are defined as colorectal adenomas occurring in patients under 50 years of age that serve as a precursor to EOCRC. By analyzing a large cohort of individuals under 50 years old who underwent screening colonoscopies, we aimed to determine whether elevated IR markers can serve as early indicators of an increased CRC risk in younger populations.

## 2. Materials and Methods

### 2.1. Study Design and Population

**Figure 1:**
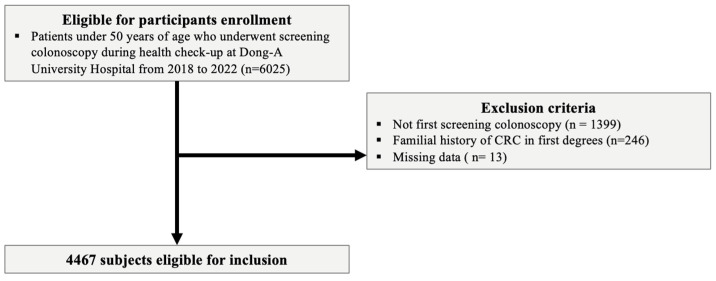
The flow of the selection of the study populations.

In this retrospective study, data from the Dong-A University Hospital Health Screening Center were analyzed. We included asymptomatic individuals aged 20 to 49 years who underwent their first colonoscopy and had concurrent measurements of FBG, TGs, high-density lipoprotein cholesterol (HDL-C), and body mass index (BMI). The study period was from 1 January 2018 to 31 December 2022. Initially, 6025 individuals under 50 years of age who underwent screening colonoscopy were considered. After excluding 1399 individuals who were not subjected to their first screening colonoscopy, 246 individuals with a familial history of CRC in first-degree relatives, and 13 individuals with missing data, 4467 subjects remained eligible for inclusion in the study (Figure 1).

### 2.2. Definitions of Variables

The equations used to quantify IR, including the TyG index, TG/HDL-C ratio, and METS-IR, were evaluated based on levels of TGs, FBG, HDL-C, and BMI. The TyG index was calculated by taking the natural logarithm (ln) using the following equation: ln[TG (mg/dL) × FBG (mg/dL)/2]. The TG/HDL-C ratio was calculated using the following equation: TG (mg/dL)/HDL-C (mg/dL). The METS-IR was calculated using the following equation: ln[(2 × FBG (mg/dL) + serum TG level (mg/dL)] × BMI (kg/m^2^)/ln [serum HDL-C level (mg/dL)].

YOAs are defined as cases where polyps found during colonoscopy in individuals under 50 years of age are resected and pathologically confirmed as tubular adenomas, villous adenomas, villotubular adenomas, or serrated lesions. Additionally, advanced YOA is characterized by polyps that are larger than 10 mm, exhibit high-grade dysplasia, or feature a villous component. FBG, HDL-C, LDL-C, and TG were assessed based on blood tests conducted on the day of the health examination, including colonoscopy. A questionnaire was used to verify whether individuals were taking medication for hypertension, diabetes, or hyperlipidemia and to check their lifestyle habits, such as alcohol consumption and smoking.

### 2.3. Primary Endpoint

The primary endpoint of this study was a comparison of the TyG index, TG/HDL-C ratio, and METS-IR values between patients with YOA and a control group consisting of individuals who underwent colonoscopy but had no evidence of adenomas.

### 2.4. Statistics and Data Analysis

Statistical analyses were conducted using SPSS software version 26.0. Continuous variables were compared using *t*-tests, and categorical variables were analyzed with chi-square or Fisher’s exact tests. Logistic regression models were used for univariate and multivariate analyses to identify factors associated with YOAs. *p*-values < 0.05 were considered statistically significant.

## 3. Results

### 3.1. Baseline Characteristics and Clinical Features

The mean age of the study population was 39.02 ± 6.32 years. Patients with YOAs were significantly older than controls, with a mean age of 41.36 ± 5.58 years, compared to 38.41 ± 6.36 years, respectively (*p* < 0.001). The proportion of males was higher in the YOA group, at 66.0%, compared to 51.9% in the control group (*p* < 0.001). The mean BMI was greater in the YOA group (24.54 ± 3.88) than in the control group (23.67 ± 5.05, *p* = 0.000). FBG levels were elevated in the YOA group (93.65 ± 18.63) compared to those in controls (89.54 ± 16.61, *p* = 0.000). HDL-C levels were lower in the YOA group (55.18 ± 13.68) than in controls (57.90 ± 13.85, *p* < 0.001), whereas LDL-C levels were higher (132.67 ± 31.34 in the YOA group vs. 125.27 ± 30.53 in controls, *p* < 0.001). The mean TG level was significantly higher in the YOA group (133.23 ± 121.44) than in the control group (109.18 ± 75.80, *p* < 0.001). Alcohol consumption and current smoking were more prevalent among patients with YOAs than among controls (51.6% vs. 46.9%, respectively, *p* = 0.011 for alcohol; 25.9% vs. 18.0%, *p* < 0.001 for smoking). Hypertension, diabetes, and dyslipidemia were also more common in the YOA group than in controls (hypertension: 9.3% vs. 4.3%, respectively, *p* = 0.000; diabetes: 2.9% vs. 1.9%, *p* = 0.053; dyslipidemia: 4.3% vs. 2.5%, *p* = 0.004). Moreover, individuals with YOA exhibited significantly higher median TyG index values (8.51± 0.71 vs. 8.32± 0.61, *p* < 0.001), TG/HDL-C ratios (2.78 ± 3.05 vs. 2.12 ± 1.85, *p* < 0.001), and METS-IR values (35.72 ± 8.37 vs. 33.44 ± 9.11, *p* < 0.001) than controls (Table 1).

### 3.2. Univariate and Multivariate Analyses of YOAs

Age was a significant predictor of YOAs in both the univariate (OR: 1.084, 95% CI: 1.070–1.098, *p* < 0.001) and multivariate analyses (OR: 1.080, 95% CI: 1.066–1.095, *p* < 0.001). Further, the male sex increased the odds of YOAs (univariate OR: 1.797, 95% CI: 1.545–2.097, *p* < 0.001; multivariate OR: 1.520, 95% CI: 1.230–1.877, *p* < 0.001). BMI was significant in the univariate analysis (OR: 1.044, 95% CI: 1.025–1.064, *p* < 0.001) but not in the multivariate model (OR: 1.003, 95% CI: 0.987–1.019, *p* = 0.740). Fasting glucose was significant in the univariate analysis (OR: 1.012, 95% CI: 1.008–1.016, *p* < 0.001) but not in the multivariate analysis (OR: 1.002, 95% CI: 0.998–1.007, *p* = 0.263). HDL-C was not significant in the multivariate model (OR: 0.996, 95% CI: 0.990–1.003, *p* = 0.253), whereas LDL-C remained significant (OR: 1.004, 95% CI: 1.002–1.006, *p* = 0.002). TG levels were significant in both univariate (OR: 1.003, 95% CI: 1.002–1.004, *p* < 0.001) and multivariate (OR: 1.001, 95% CI: 1.000–1.002, *p* = 0.041) analyses. Alcohol consumption was not significant in the multivariate analysis (OR: 0.959, 95% CI: 0.813–1.132, *p* = 0.625), and current smoking approached significance (OR: 1.218, 95% CI: 0.982–1.511, *p* = 0.072). Hypertension remained significant in the multivariate model (OR: 1.458, 95% CI: 1.086–1.957, *p* = 0.012), whereas dyslipidemia did not (OR: 1.098, 95% CI: 0.739–1.633, *p* = 0.643) (Table 2).

The TyG index was significant in the univariate analysis (OR: 1.552, 95% CI: 1.389–1.733, *p* < 0.001) and remained significant in the multivariate model (OR: 1.064, 95% CI: 1.023–1.325, *p* = 0.021). The TG/HDL-C ratio was significant in both the univariate (OR: 1.131, 95% CI: *p* = 0.000) and multivariate analyses (OR: 1.067, 95% CI: *p* = 0.000). The METS-IR was also significant in the univariate analysis (OR: 1.031, 95% CI: 1.031–1.105, *p* = 0.000) and remained significant in the multivariate model (OR: 1.011, 95% CI: 1.002–1.021, *p* = 0.023) (Table 3).

### 3.3. Univariate and Multivariate Analyses of Advanced YOAs

Patients with advanced YOAs were older than controls (41.49 ± 5.27 vs. 38.99 ± 6.32, respectively, *p* = 0.004) and included a higher percentage of males (73.3% vs. 54.6%, *p* = 0.012). The mean BMI was higher in the advanced YOA group (25.29 ± 3.61) than in controls (23.84 ± 4.85, *p* = 0.046). Fasting glucose levels were elevated in the advanced YOA group compared to those in controls (96.16 ± 20.01 vs. 90.33 ± 17.09, respectively, *p* = 0.023), whereas HDL-C levels were lower (52.80 ± 15.22 vs. 57.39 ± 13.84, *p* = 0.027). Although LDL-C levels were higher in the advanced YOA group than in controls, this difference was not statistically significant (133.64 ± 29.85 vs. 126.73 ± 30.85, respectively, *p* = 0.134). TG levels were higher in the advanced YOA group than in controls (164.53 ± 199.17 vs. 113.62 ± 85.73, respectively, *p* = 0.094). Furthermore, hypertension and dyslipidemia were more prevalent in the advanced YOA group than in controls (hypertension: 15.6% vs. 5.2%, respectively, *p* = 0.002; dyslipidemia: 8.9% vs. 2.8%, *p* = 0.041). In addition, the TyG index and METS-IR values were significantly higher in the advanced YOA group than in the control group (8.62 ± 0.85 vs. 8.36 ± 0.63, respectively, *p* < 0.45 for the TyG index; 37.70 ± 8.49 vs. 33.87 ± 9.01, *p* = 0.005 for METS IR), whereas results for the TG/HDL-C ratio were not significant (3.69 ± 5.22 vs. 2.24 ± 2.11, *p* = 0.069) (Table 4).

The TyG index was significant in the univariate analysis (OR: 1.769, 95% CI: 1.177–2.659, *p* = 0.006) but not in the multivariate model (OR: 1.288, 95% CI: 0.804–2.064, *p* = 0.292). The TG/HDL-C ratio was significant in both the univariate (OR: 1.120, 95% CI: 1.053–1.191, *p* < 0.001) and multivariate (OR: 1.078, 95%CI: 1.006–1.155, *p* = 0.032) analyses. Meanwhile, the METS-IR was significant in the univariate analysis (OR: 1.014, 95%CI: 1.002–1.027, *p* = 0.022) but not in the multivariate model (OR: 1.009, 95% CI: 0.994–1.025, *p* = 0.245) (Table 5).

## 4. Discussion

The findings of our study underscore the significant association between elevated surrogate markers of IR and the presence of YOAs in individuals under 50 years old. Specifically, higher TyG index, TG/HDL-C ratio, and METS-IR values were strongly associated with an increased incidence of YOAs, even after adjusting for potential confounders such as age, sex, BMI, and lifestyle factors (e.g., smoking and alcohol consumption). These findings suggest that metabolic dysregulation might play an important role in YOAs, potentially serving as a precursor to EOCRC.

The correlation between metabolic disorders and CRC is well documented, and similar associations have been observed in EOCRC. Several studies analyzing risk factors of EOCRC have demonstrated that an increased BMI and the presence of MetS contribute to a higher risk of developing this form of cancer [14,16,17]. Several mechanisms contribute to CRC development due to obesity or MetS. Adipose tissue secretes inflammatory cytokines that cause chronic inflammation [18,19]. Changes in sex hormones and alterations in the gut microbiome are also involved [20,21]. IR serves as an important factor by stimulating hyperinsulinemia, which then triggers the phosphoinositide 3-kinase/protein kinase B/mammalian target of rapamycin pathway in patients with CRC. The activation of this pathway elevates insulin-like growth factor-1 levels and alters signaling through peroxisome proliferator-activated receptor gamma and nuclear factor kappa B, contributing to CRC progression [22,23,24]. Whereas the hyperinsulinemic–euglycemic clamp is recognized as the gold standard for IR diagnoses, its practical application in clinical settings is limited owing to its complexity. This has led to the increased use of surrogate markers as alternatives for assessing IR. Key surrogate markers of IR include the TyG index, TG/HDL-C ratio, and METS-IR, as these indicators have been shown to be associated with CRC [8,9,10,11]. Furthermore, in a retrospective study, a significant correlation between the TyG index and colorectal adenomas, a precursor to CRC, was reported [25].

Owing to the increase in EOCRC prevalence, the U.S. Preventive Services Task Force has updated its guidelines, lowering the recommended age for initiating CRC screenings via colonoscopy from 50 to 45 years [26]. Despite this, perfect prevention remains unachievable. Individuals under 50 years old with advanced YOAs have an 8-fold increased risk of developing CRC compared to those with normal colonoscopy results [27]. This suggests that early detection and appropriate follow-up of YOAs can reduce the incidence of CRC. Therefore, it is essential to identify and analyze risk factors of EOCRC and its precursors to establish personalized screening timelines.

Simplified IR markers could contribute to determining these individualized screening schedules, suggesting further research is needed in this area. These markers could serve as crucial early indicators of YOA risks, highlighting the need for targeted metabolic screening during routine colonoscopy in younger adults. Such proactive measures could enable early interventions, potentially impeding the progression from adenoma to carcinoma, particularly in individuals with a predisposition to MetS. Moreover, the increase in metabolic disorders, including obesity and diabetes, underscores the importance of addressing these modifiable risk factors through lifestyle changes or medication. Future research should explore whether interventions aimed at modifying these risk factors could effectively reduce the incidence of YOA and, by extension, EOCRC.

This study had several limitations. First, it was conducted at a single institution, analyzing risk factors and IR markers for YOAs. Therefore, the analysis was limited by the small number of cases of advanced YOAs, which have a higher risk of developing into CRC. Moreover, this was a retrospective cross-sectional study that did not involve a longitudinal cohort analysis, limiting its temporal scope. Additionally, the study was focused solely on Koreans, and therefore, its applicability to other ethnicities remains uncertain. Moreover, data on dietary habits, such as red meat consumption, were unavailable, which could act as confounders affecting the results. Despite these limitations, this research was the first to analyze the correlation between IR markers and YOA. In South Korea, where access to healthcare is relatively good, many individuals under 50 years undergo colonoscopies, allowing for a comprehensive analysis of related factors. Our study suggests that surrogate markers of IR could serve as early indicators of an increased CRC risk in younger populations, highlighting the potential for more personalized and effective screening strategies. Further research is warranted to validate these findings and to explore the implementation of IR markers in routine clinical practice for CRC prevention. Larger, multi-center longitudinal studies are also necessary to validate these findings.

## 5. Conclusions

In conclusion, elevated markers of IR are strongly associated with the occurrence of YOAs in individuals under 50 years old. These findings suggest that surrogate markers of IR might represent early indicators of an increased CRC risk in younger populations.

## Figures and Tables

**Table 1:** Baseline characteristics of the study population and differences in clinical characteristics of patients with adenoma and patients without adenoma.

Characteristics	All Patients (*n* = 4467)	Control (*n* = 3547)	Patients with YOA (*n* = 920)	*p* Value
Age (mean ± SD)	39.02 ± 6.32	38.41 ± 6.36	41.36 ± 5.58	<0.001
Gender male, *n* (%)	2448 (54.8)	1841 (51.9)	607 (66.0)	<0.001
BMI (mean ± SD)	23.85 ± 4.84	23.67 ± 5.05	24.54 ± 3.88	<0.001
Fasting glucose (mean ± SD)	90.39 ± 17.13	89.54 ± 16.61	93.65 ± 18.63	<0.001
HDL-C (mean ± SD)	57.34 ± 13.86	57.90 ± 13.85	55.18 ± 13.68	<0.001
LDL-C (mean ± SD)	126.79 ± 30.84	125.27 ± 30.53	132.67 ± 31.34	<0.001
TG (mean ± SD)	114.13 ± 87.70	109.18 ± 75.80	133.23 ± 121.44	<0.001
Alcohol consumption, *n* (%)	2140 (47.9)	1665 (46.9)	475 (51.6)	0.011
Current smoker, *n* (%)	878 (19.7)	640 (18.0)	238 (25.9)	<0.001
Medical History				
Hypertension, *n* (%)	238 (5.3)	152 (4.3)	86 (9.3)	<0.001
Diabetes, *n* (%)	94 (2.1)	67 (1.9)	27 (2.9)	0.053
Dyslipidemia, *n* (%)	130 (2.9)	90 (2.5)	40 (4.3)	0.004
TyG index (mean ± SD)	8.36 ± 0.64	8.32 ± 0.61	8.51 ± 0.71	<0.001
TG/HDL-C (mean ± SD)	2.25 ± 2.17	2.12 ± 1.85	2.78 ± 3.05	<0.001
METS-IR (mean ± SD)	33.91 ± 9.01	33.44 ± 9.11	35.72 ± 8.37	<0.001

SD: standard deviation.

**Table 2:** Univariate and multivariate analyses related to young-onset colorectal adenoma.

Characteristics	Univariate		Multivariate	
	OR	*p* Value	OR	*p* Value
Age	1.084 (1.070–1.098)	0.000	1.080 (1.066–1.095)	0.000
Gender Male	1.797 (1.545–2.097)	0.000	1.520 (1.230–1.877)	0.000
BMI	1.044 (1.025–1.064)	0.000	1.003 (0.987–1.019)	0.740
Fasting Glucose	1.012 (1.008–1.016)	0.000	1.002 (0.998–1.007)	0.263
HDL-C	0.985 (0.980–0.991)	0.000	0.996 (0.990–1.003)	0.253
LDL-C	1.008 (1.005–1.010)	0.000	1.004 (1.002–1.006)	0.002
TG	1.003 (1.002–1.004)	0.000	1.001 (1.000–1.002)	0.041
Alcohol consumption	1.207 (1.044–1.395)	0.011	0.959 (0.813–1.132)	0.625
Smoker		0.000		0.152
Ex-smoker	1.510 (1.249–1.825)	0.000	1.017 (0.815–1.268)	0.883
Current smoker	1.752 (1.466–2.093)	0.000	1.218 (0.982–1.511)	0.072
Hypertension	2.303 (1.749–3.033)	0.000	1.458 (1.086–1.957)	0.012
Diabetes	1.570 (0.999–2.470)	0.051		
Dyslipidemia	1.746 (1.194–2.552)	0.004	1.098 (0.739–1.633)	0.643

**Table 3:** Odds ratios and 95% confidence intervals for the incidence of young-onset colorectal adenoma according to the TyG index, TG/HDL ratio, and METS-IR using logistic regression analysis.

Characteristics	Univariate		Multivariate	
	OR	*p* Value	OR	*p* Value
TyG index	1.552 (1.389–1.733)	0.000	1.064 (1.023–1.325)	0.021
TG/HDL-C	1.131 (1.096–1.168)	0.000	1.067 (1.031–1.105)	0.000
METS-IR	1.031 (1.022–1.041)	0.000	1.011 (1.002–1.021)	0.023

**Table 4:** Baseline characteristics of the study population and differences in clinical characteristics of patients with advanced young-onset colorectal adenoma.

Characteristics	All Patients (*n* = 4467)	Control (*n* = 4422)	Patients with Advanced YOA (*n* = 45)	*p* Value
Age (mean ± SD)	39.02 ± 6.32	38.99 ± 6.32	41.49 ± 5.27	0.004
Gender male, *n* (%)	2448 (54.8)	2415 (54.6)	33 (73.3)	0.012
BMI (mean ± SD)	23.85 ± 4.84	23.84 ± 4.85	25.29 ± 3.61	0.046
Fasting glucose (mean ± SD)	90.39 ± 17.13	90.33 ± 17.09	96.16 ± 20.01	0.023
HDL-C (mean ± SD)	57.34 ± 13.86	57.39 ± 13.84	52.80 ± 15.22	0.027
LDL-C (mean ± SD)	126.79 ± 30.84	126.73 ± 30.85	133.64 ± 29.85	0.134
TG (mean ± SD)	114.13 ± 87.70	113.62 ± 85.73	164.53 ± 199.17	0.094
Alcohol consumption, *n* (%)	2140 (47.9)	2119 (47.9)	21 (46.7)	0.882
Current smoker, *n* (%)	878 (19.7)	864 (19.3)	14 (31.1)	0.052
Medical History				
Hypertension, *n* (%)	238 (5.3)	231 (5.2)	7 (15.6)	0.002
Diabetes, *n* (%)	94 (2.1)	93 (2.1)	1 (2.2)	0.618
Dyslipidemia, *n* (%)	130 (2.9)	126 (2.8)	4 (8.9)	0.041
TyG index (mean ± SD)	8.36 ± 0.64	8.36 ± 0.63	8.62 ± 0.85	0.045
TG/HDL-C (mean ± SD)	2.25 ± 2.17	2.24 ± 2.11	3.69 ± 5.22	0.069
METS-IR (mean ± SD)	33.91 ± 9.01	33.87 ± 9.01	37.70 ± 8.49	0.005

SD: standard deviation.

**Table 5:** Odds ratios and 95% confidence intervals for the incidence of young-onset colorectal advanced adenoma according to the TyG index, TG/HDL ratio, and METS-IR using logistic regression analysis.

Characteristics	Univariate		Multivariate	
	OR	*p* Value	OR	*p* Value
TyG	1.769 (1.177–2.659)	0.006	1.288 (0.804–2.064)	0.292
TG/HDL-C	1.120 (1.053–1.191)	0.000	1.078 (1.006–1.155)	0.032
METS-IR	1.014 (1.002–1.027)	0.022	1.009 (0.994–1.025)	0.245

## Data Availability

The data presented in this study are available on request from the corresponding author.
